# Supplementary material for: Lipidomic Profile of Human Sperm Membrane Identifies a Clustering of Lipids Associated with Semen Quality and Function
Source: Int J Mol Sci. 2023 Dec 25;25(1):297. doi: 10.3390/ijms25010297 (PMC10778809; doi:10.3390/ijms25010297)
Supplement: Supplementary file 1 [file ijms-25-00297-s001.zip › ijms-2777346-supplementary Figure S1.pdf]

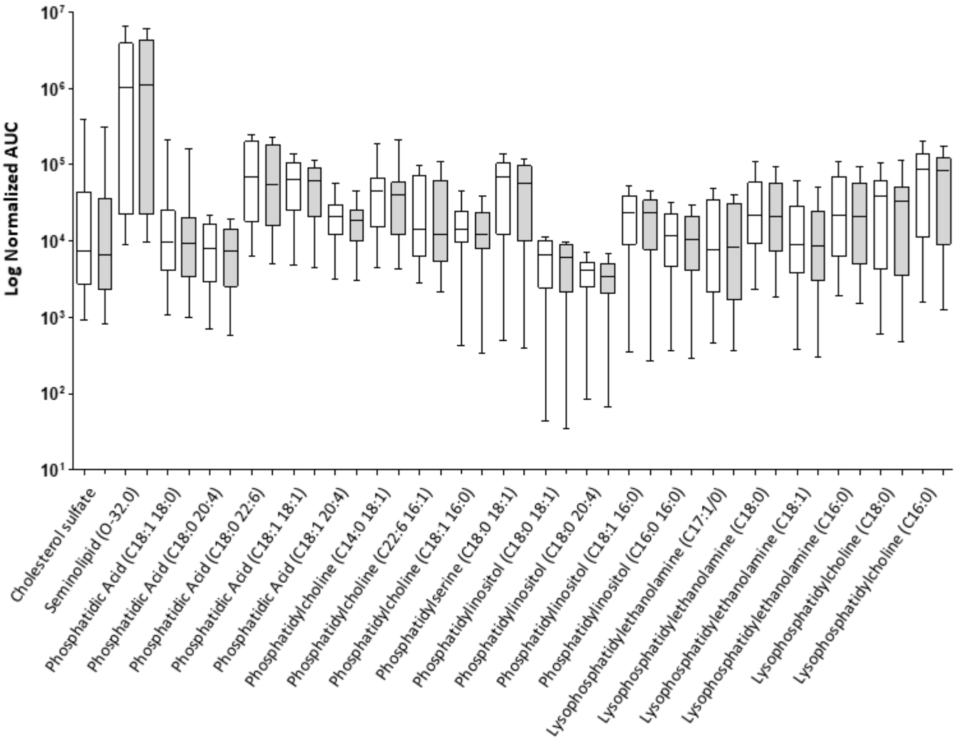

Supplemental Figure S1. Comparative evaluation of relative standardized lipid composition measured as logarithmic AUC between pelleted samples (white bars) and density gradient isolated sperm cells (grey bars) within a subpopulation of patients (N = 10).
